# Combined genetic and chemical screens indicate protective potential for EGFR inhibition to cardiomyocytes under hypoxia

**DOI:** 10.1038/s41598-021-96033-z

**Published:** 2021-08-17

**Authors:** Juho Heliste, Anne Jokilammi, Katri Vaparanta, Ilkka Paatero, Klaus Elenius

**Affiliations:** 1 Institute of Biomedicine, University of Turku, Kiinamyllynkatu 10, 20014 Turku, Finland; 2 Turku Doctoral Programme of Molecular Medicine, University of Turku, Turku, Finland; 3 Turku Bioscience Centre, University of Turku and Åbo Akademi University, Tykistökatu 6B, 20520 Turku, Finland; 4 MediCity Research Laboratories, University of Turku, Tykistökatu 6A, 20520 Turku, Finland; 5 Department of Oncology, Turku University Hospital, PO Box 52, 20521 Turku, Finland

**Keywords:** Cardiovascular diseases, Molecular medicine, Myocardial infarction

## Abstract

The return of blood flow to ischemic heart after myocardial infarction causes ischemia–reperfusion injury. There is a clinical need for novel therapeutic targets to treat myocardial ischemia–reperfusion injury. Here we screened for targets for the treatment of ischemia–reperfusion injury using a combination of shRNA and drug library analyses in HL-1 mouse cardiomyocytes subjected to hypoxia and reoxygenation. The shRNA library included lentiviral constructs targeting 4625 genes and the drug library 689 chemical compounds approved by the Food and Drug Administration (FDA). Data were analyzed using protein–protein interaction and pathway analyses. EGFR inhibition was identified as a cardioprotective mechanism in both approaches. Inhibition of EGFR kinase activity with gefitinib improved cardiomyocyte viability in vitro. In addition, gefitinib preserved cardiac contractility in zebrafish embryos exposed to hypoxia-reoxygenation in vivo. These findings indicate that the EGFR inhibitor gefitinib is a potential candidate for further studies of repurposing the drug for the treatment of myocardial infarction.

## Introduction

Despite advances in pharmacological and invasive therapies for acute myocardial infarction, the damage caused by ischemia and subsequent reperfusion still frequently leads to significant loss of myocardial function and heart failure^1^. Numerous efforts to pharmacologically promote the survival of myocardium during and after ischemia have been made. These include approaches to treat patients with acute myocardial infarction with e.g. glucose–insulin–potassium^2^, exenatide^3,4^, adenosine^5^, or metoprolol^6^. However, no effective drugs that would specifically target the mechanisms of ischemia–reperfusion are currently in clinical use. Improved understanding of the underlying mechanisms is expected to enhance the development of novel therapeutic approaches^7^.

High-throughput screening platforms taking advantage of drug libraries have been widely used to identify therapeutic targets for cancer^8^. In contrast, relatively few attempts have been made to exploit these methods to characterize molecular mechanisms regulating the pathogenesis of heart diseases. The few examples include the use of rat cardiomyoblastic H9c2 cells to screen for compounds protecting from doxorubicin-induced cardiotoxicity^9^, or stem cell-derived human cardiomyocytes to address cardiotoxicities related to tyrosine kinase inhibitors (TKI)^10^. The H9c2 cells have also been previously used in a large screen of cardioprotective compounds in a cell-based model of ischemia–reperfusion^11^. Similarly, genome-wide functional screens with gene silencing techniques such as short hairpin RNAs (shRNA) have been used to detect therapeutic targets in cancer models^12,13^, but their use to identify druggable targets for cardiac diseases has been limited.

Here, we used a combination of two functional screens, one based on an shRNA library targeting 4625 genes and the other on a chemical library including 689 FDA-approved drugs, to screen for targets of myocardial ischemia–reperfusion injury using HL-1 cardiomyocytes exposed to hypoxia and reoxygenation. Protein–protein interaction and pathway analyses of the top hits from both screens suggested EGFR inhibition as a potential mechanism to enhance the survival of hypoxic cardiomyocytes. Consistently, the EGFR TKI gefitinib promoted survival of cardiomyocytes in vitro as well as rescued the contractility of the hearts of zebrafish embryos in vivo under hypoxic conditions. EGFR inhibitors may thus represent a novel approach to be investigated for the treatment of myocardial ischemia–reperfusion injury.

## Materials and methods

### Cell culture

HL-1 mouse atrial cardiomyocytes were a kind gift from Dr. Pasi Tavi (University of Eastern Finland). Cells were cultured in Claycomb medium (Sigma), supplemented with 10% fetal bovine serum, 50 U/ml of both penicillin and streptomycin, 2 mM l-glutamine or UltraGlutamine (Lonza), and 0.1 mM norepinephrine. Cells were maintained in an atmosphere of 5% CO_2_ and 21% O_2_ with humidity of 95% and temperature of 37 °C. Cells were passaged every 3 or 4 days when they reached confluency. Culture plates were coated with a solution of 0.02% gelatin and 10 μg/ml fibronectin (R&D Systems) for at least an hour at 37 °C.

### CTG cell viability assays

Cellular viability was measured with CellTiter-Glo (CTG) assays (Promega) according to manufacturer’s instructions. To address the effect of EGFR inhibition on cell viability in the validation experiments, cells were plated on 96-well plates at 5000 cells per well. Cells were treated with 1.6–1000 ng/ml of gefitinib in 0.01% DMSO (Cayman Chemicals) for 72 h starting at the moment of plating. DMSO-treated cells were used as controls.

### Hypoxia and reoxygenation

Hypoxic treatments for cell viability measurements were carried out for 24–48 h using a hypoxic workstation (InVivo2, Ruskinn Technology Ltd.) with 1% O_2_ concentration. For Western analyses, a shorter period of 3 h in hypoxia was used to detect short-term regulation in protein phosphorylation status. For the shRNA screening (see details below), the cells were deoxygenated for 1 h and subsequently placed into a deoxygenated plastic bag (in the hypoxic chamber) and kept in this sealed hypoxic environment in a normal cell incubator for 96 h. This longer period was chosen to ensure robust cell death and differences in shRNA abundances. For reoxygenation, the cells were returned to normal cell incubator with 21% O_2_.

### ShRNA library screening

HL-1 cells were lentivirally infected with DECIPHER Mouse Module 1 shRNA library (Cellecta) according to manufacturer’s instructions in three separate transductions. The library targets 4625 genes with up to six separate barcoded shRNAs per gene (total of 27,500 shRNAs). Infected populations were split to two, half of the cells serving as control populations and half being exposed to hypoxia-reoxygenation. The cells were deoxygenated as described above and were subsequently rexoygenated for 3 h. Control populations were grown in a normal cell incubator for the same time as the hypoxia-reoxygenation-treated cells. Cells were trypsinized, pelleted, and stored in − 80 °C until DNA extraction with NucleoSpin DNA extraction kit (Macherey–Nagel). The three separate samples from both control and hypoxia-reoxygenation populations were pooled and sequenced at Cellecta using next generation sequencing. Counts of raw reads and reads normalized to 20 million reads within each sample were analyzed for each shRNA. Data for each shRNA were additionally normalized against the expected count of reads specific for the given shRNA plasmid in the original library used for the infection.

In the statistical analyses, separate shRNAs per gene were used as internal replicates. The mean rank in three independent statistical tests (nonparametric quantile regression^14^, DESeq2^15^ with separate shRNAs for the same target as covariates, and NBPSeq^16^) was used as a measure of significance for differential expression analysis with a cutoff at mean rank of 500.

### Drug library screening

Drug library screening using 689 FDA-approved compounds (Supplementary Dataset S1) was bought as a service from Misvik Biology Inc. (Turku, Finland). Cells were plated on 384-well plates at a density of 1000 cells per well together with the drugs at concentrations of 40, 200, 1000, or 5000 nM in 0.01% DMSO. DMSO-treated cells were used as controls. One day after plating, the cells were moved to hypoxic workstation for 48 h and subsequently reoxygenated in a normal cell incubator for 24 h. Cells grown in normoxia for the same time were used as controls. Cellular viability was measured with the CTG assay. Data were normalized with values obtained from DMSO-treated control cells.

The relative luminescence unit (RLU) value of the drug concentration with the highest RLU value in the hypoxia-reoxygenation treatment compared to the normoxic control was used as a representative value. Drugs associated with representative values over one standard deviation above the value in the DMSO-treated control in hypoxia-reoxygenation treatment were considered significant in inducing cell survival in the hypoxia-reoxygenation condition.

### Protein–protein interaction analysis and pathway analysis

The protein targets of the drug screen compounds were acquired from the DrugBank database^17^. The drug targets that were not present in the DrugBank were manually annotated from literature or from PubChem. The drugs were categorized into agonists (including terms: "potentiator", "activator", "agonist", "inducer", "ligand", "partial agonist", "positive allosteric modulator", "stimulator") or antagonists (including terms: "antagonist", "antisense", "antisense oligonucleotide", "blocker", "competitive", "inhibitor", "inhibitory allosteric modulator", "inverse agonist", "negative modulator", "partial antagonist", "suppressor", "vaccine") according to the Drug Gene Interaction Database using rDGIdb wrapper^18,19^ to access the database with R. Experimentally validated protein–protein interactions were downloaded from the PSICQUIC webservice through Bioconductor PSICQUIC R wrapper^20^.

The protein–protein interactions were superimposed on the list of genes with significantly upregulated or downregulated shRNAs or the targets of the categorized agonists or antagonists that induced survival in the drug screen to form a network graph. In the network graphs the nodes were chosen to represent proteins and the edges protein–protein interactions. The most connected (high degree) nodes were considered as significant central regulators among the list of proteins.

For pathway analysis, all available pathway annotations from Molecular Signatures Database v.6.0^21^ were acquired. Only protein targets of drugs that induced survival in the drug screen were included. For the shRNA screen pathway analysis, the median normalized count of independent shRNAs targeting the same protein in both treatments were calculated. The targets of shRNAs that were enriched by at least 30% in the hypoxia-reoxygenation treatment according to the median normalized counts were included. The dimensionality reduced values for a pathway in control treatment and hypoxia-reoxygenation treatment were calculated with maximally collapsing metric learning (MCML)^22,23^ from median normalized shRNA counts or normalized representative RLU values. The difference between the dimensionality reduced pathway values for control and hypoxia-reoxygenation treatments were used as a pathway significance score (the smaller the more significant for the drug screen and the higher the more significant for the shRNA screen).

The statistical significance of a pathway score was calculated by randomly choosing 10,000 protein sets of the same length from the data and fitting a probability distribution function with an Epanechnikov kernel function to the pathway scores calculated from the randomized protein sets. The *P* value was inferred from the cumulative distribution function of the pathway scores. Additionally, a statistical test was devised to calculate the statistical significance of the fitness of each pathway to the data, since whole *mus musculus* proteome was not represented in the drug screen or shRNA screen data. The statistical significance of pathway fitness was calculated by randomly choosing 100 protein sets of same length as the protein set of the targets of the drugs that induced survival in the hypoxia-reoxygenation condition from all the drug targets. For the shRNA screen data, 10 protein sets of same length as the shRNA targets that were enriched in the hypoxia-reoxygenation treatment were randomly chosen from all proteins represented in the pathway annotations. The occupancy of the randomized protein sets in each pathway annotation was calculated by dividing the number of found proteins of the pathway in the protein set by the number of proteins in the pathway annotation. A probability distribution function was fitted with an Epanechnikov kernel function to the occupancy values from randomized protein sets and the *P* value was inferred from the cumulative distribution function of the occupancy values. Both Matlab R2016b^24^ and R^25^ were used for the analysis.

### Zebrafish embryo model of hypoxia-reoxygenation

Analyses of zebrafish embryos were carried out under the licenses MMM/465/712-93 (issued by the Finnish Ministry of Forestry and Agricultur) and ESAVI/9339/04.10.07/2016 (granted by Project Authorisation Board of Regional State Administrative Agency for Southern Finland) according to the regulations of the Finnish Act on Animal Experimentation (62/2006). Study was carried out in compliance with the ARRIVE guidelines. Zebrafish embryos of casper strain^26^ were obtained by using natural spawning of the fish in the breeding tanks. There was no exclusion of embryos and collection and analyses were performed in a non-blinded manner. The embryos were randomly harvested as a batch from the tank. The same person (J.H.) performed the microscopy and video analysis, while another (I.P.) performed the actual experimentation with the embryos. The embryos were cultured in E3 medium at 28.5 °C until subjected to hypoxia-reoxygenation 3 days after fertilization. The embryos were treated with gefitinib or DMSO (1%) for 1 h prior to hypoxia. Hypoxic medium was produced by boiling E3 medium for 5 min, adding 1 mg/ml of sodium sulphite as oxygen scavenger^27^, dispensing hot medium into 30 ml glass vials, cooling the solution in a 28.5 °C water bath, and neutralizing pH to 7. Once the hypoxic medium was equilibrated to 28.5 °C, the embryos were slowly transferred with a glass pipette to glass vials containing hypoxic medium. After 15 or 30 min of incubation in the hypoxic medium, the embryos were transferred to petri dishes with normoxic E3 medium, then washed with normoxic E3 and finally transferred to normoxic E3 medium containing gefitinib or DMSO (1%) and incubated at 28.5 °C until analyzed the next day. Five to 10-s movies of the embryos were filmed using a Zeiss StereoLumar stereomicroscope (at 11 frames per second). Heart rate and ejection fraction were measured manually using Fiji^28,29^.

Level of dissolved oxygen was controlled using semiquantitative colorimetric JBL PROAQUATEST O2 Oxygen test kit (JBL GmbH & Co. KG) with three technical replicates. All replicates produced similar results. Normoxic E3 medium contained 8 mg/l of dissolved oxygen. After boiling and sodium disulfite treatment the dissolved oxygen content was reduced to < 0.2 mg/l and remained at < 0.2 mg/l for 30 min after the oxygen removal.

### Whole-mount immunofluorescence analysis

Zebrafish embryos were collected after 15 min of hypoxia followed by 24-h reoxygenation and fixed with 4% paraformaldehyde/PBSTw (PBS, 0.2% Tween-20) for overnight at + 4 °C. After fixation, the samples were stored in PBSTw until processed further. For immunostaining, the samples were permabilized using cold methanol (− 20 °C for 20 min), followed by incubation in cold acetone (− 20 °C for 30 min). The samples were washed with PBSTw and non-specific binding blocked by an overnight incubation in blocking solution (PBSTw, 5% FCS). To visualize myocardium, the samples were incubated in the presence of anti-tropomyosin (Developmental Studies Hybridoma Bank (DSHB) Product CH1; deposited to the DSHB by Lin, J.J.-C.) for 3 days at + 4 °C. After six 30 min washes at room temperature with PBSTw, the samples were incubated with donkey anti-mouse-Alexa647 (ThermoFisher Scientific) secondary antibody and the nuclear dye 4′,6-diamidino-2-phenylindole (DAPI) for 3 days at + 4 °C. After six 30 min washes at room temperature, the samples were incubated in PBSTw at + 4 °C overnight until mounted on glass-bottom dishes using 0.7% low-melting point agarose and overlaid with PBSTw. The whole mount samples were imaged at room temperature using 3i Marianas CSU-W1 spinning disk microscope equipped with 40×/NA1.1 water immersion objective, 405 nm and 640 nm lasers, and emission filters (DAPI , 445/45 nm; Cy5/Alexa647, 692/40 nm). Images were collected using Hamamatsu sCMOS Orca Flash4.0, 2 × 2 binning with final x–y resolution of 0.317 µm and 16-bit depth. One central optical section of the ventricle was imaged from each embryo. The images were analyzed using FIJI. The background was subtracted using rolling ball radius of 50. The brightness and contrast were linearly adjusted prior to measurements, and each image and channel was processed separately to ensure optimal representation of morphological features. To measure the average thickness of the myocardium, first the ventricle was outlined using segmented line tool and the area was measured (*A*_*out*_). Next, the inside area of the ventricle was similarly measured (*A*_*in*_). Finally, the average thickness of myocardium was calculated using following equation (Eq. 1).

1 $${myocardium\;thickness}= \sqrt{\frac{{A}_{out}}{\pi }}-\sqrt{\frac{{A}_{in}}{\pi }}$$

Statistical analysis of the data was carried out using GraphPad Prism (6.0) software and one-way ANOVA with Tukey post-hoc test.

### Western analysis

For Western blot analyses, HL-1 cells were grown on 6-well plate wells (300,000 cells/well), treated overnight with 100 nM gefitinib and exposed to hypoxia for 3 h. Cells were lysed with lysis buffer^30^, and 30 or 60 μg of extracted total protein was separated with gel electroforesis on 4–15% Criterion TGX precast gels (Bio-Rad) and transferred to nitrocellulose membranes. Membranes were blocked for 1 h in blocking solution (5% non-fat milk or bovine serum albumin in 10 mM Tris–HCl (pH 7.4), 150 mM NaCl and 0.05% Tween-20). Primary antibodies (anti-phosphorylated Akt (S473) (catalog number #4060S, Cell Signaling Technology), anti-Akt (#2920S, Cell Signaling Technology), anti-phosphorylated ERK (Y204/Y187) (#5726S, Cell Signaling Technology), anti-ERK (#9102S, Cell Signaling Technology), anti-phosphorylated p38 (T180/Y182) (#9211S, Cell Signaling Technology), anti-p38 (sc-7149, Santa Cruz Biotechnology), anti-β-tubulin (sc-9104, Santa Cruz Biotechnology), and anti-actin (sc-1616, Santa Cruz Biotechnology)) were incubated overnight with membranes in blocking solution at 4 °C. Incubations with secondary antibodies (Santa Cruz Biotechnology) were performed for 1 h at room temperature. Immunosignals were detected with WesternBright ECL HRP substrate reagent (Advansta) and imaged with ImageQuant LAS 4000 (GE Healthcare Life Sciences). Densitometric analysis was performed with NIH ImageJ v1.50i software.

### Statistical analyses

Statistical analyses of data from shRNA and drug screenings are described above. Significance of differences between treatments in validation experiments with cardiomyocytes was analyzed with one-way ANOVA for multiple group comparisons, and pairwise differences were tested with paired, two-tailed Student’s t tests. False discovery rate (FDR) method^31^ was used for correction of *P* values for multiple testing. For zebrafish embryo experiments, Kruskal–Wallis test followed by Mann–Whitney U test, or one-way ANOVA followed by Student’s t test, and subsequent FDR-correction of *P* values was used. Data are presented as boxplots (red horizontal lines representing median, box representing first and third quartile, and whiskers representing the range of the data) or as dotplots. Heatmaps were generated with pheatmap package^32^ for R. All statistical analyses were performed with RStudio^33^.

## Results

### ShRNA library screen

**Figure 1:**
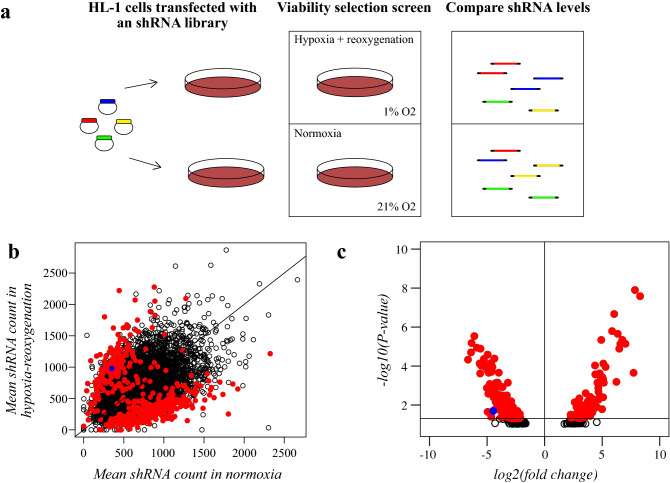
shRNA library screening to identify modulators of cardiomyocyte survival. (**a**) Schematic overview of the shRNA library screen. HL-1 cells were transduced with a lentiviral shRNA library targeting 4625 genes. Cells were exposed to hypoxia-reoxygenation for a viability dropout screen. As the readout, shRNA abundances were quantified with NGS. (**b**) Scatter plot illustrating the mean copy number of barcoded shRNAs for each gene after culturing of the cells in normoxia (x-axis) or in hypoxia followed by reoxygenation (y-axis). Significantly regulated genes are depicted with red dots. The dot representing the mean copy number of shRNAs targeting *EGFR* is indicated in blue. Line depicts the level where shRNA abundances are equal in normoxia and hypoxia-reoxygenation. (**c**) Volcano plot of log2-transformed fold-changes of mean shRNA counts in normoxic cells and in hypoxia-reoxygenation-treated cells and their respective *P* values from DESeq2 analysis. Significantly regulated genes are depicted with red dots. The dot representing *EGFR* is indicated in blue.

To identify genes that functionally regulate survival of cardiomyocytes during hypoxia-reoxygenation, HL-1 cells were infected with a lentiviral library of barcoded shRNAs targeting 4625 genes (Fig. 1a). Each of the genes was targeted with one to 18 shRNAs, with a median of six shRNAs per gene. The cells were subjected into a dropout screening approach, in which the enrichment or depletion of individual shRNA species in the cells surviving the hypoxia-reoxygenation treatment was assessed by comparing to quantities of the same shRNAs in cells cultured in normoxia. Abundances of genome-integrated shRNAs were detected with NGS. The means of copy numbers of the shRNAs for each gene in normoxia and after hypoxia-reoxygenation are presented as a scatterplot in Fig. 1b. Copy numbers of each barcoded shRNA for each gene are listed in Supplementary Dataset S2.

According to the experimental design, shRNAs targeting genes that suppress cardiomyocyte survival were expected to become enriched, whereas shRNAs targeting genes that improve cardiomyocyte survival were expected to be depleted in the hypoxia-reoxygenation-treated population, as compared to control cells. The analysis identified 158 genes with significantly enriched shRNAs and 262 genes with significantly depleted shRNAs in hypoxia-reoxygenation (Supplementary Fig. S1). Genes with mean rank < 500 were defined as significantly regulated (see “Materials and methods” for details). Genes with significantly regulated shRNAs are depicted as red dots in Fig. 1b. Additionally, log2-transformed fold changes of shRNA abundances in hypoxia-reoxygenation vs. normoxia are presented as a volcano plot, where y-axis depicts the adjusted *P* values from differential expression analysis with DESeq2 algorithm (Fig. 1c).

### Drug library screen

**Figure 2:**
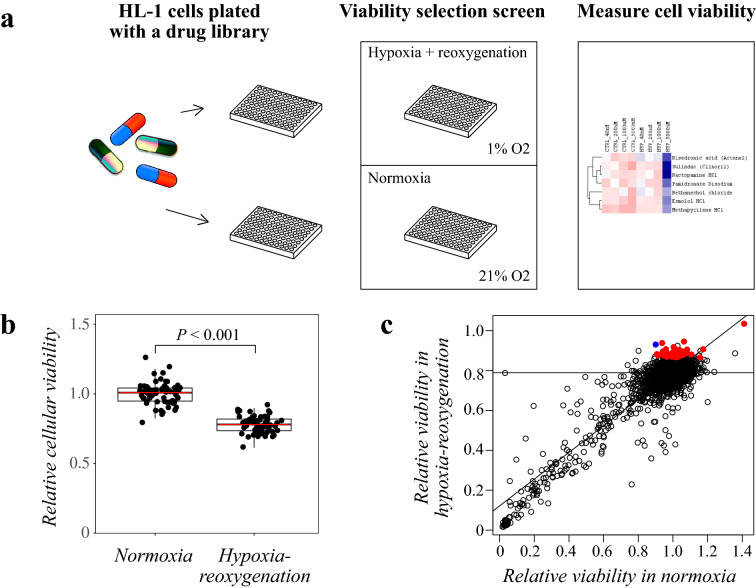
Drug library screening to identify compounds affecting cardiomyocyte viability. Schematic overview of the drug library screen. HL-1 cells were plated with a drug library consisting of 689 FDA-approved compounds. Cells were exposed to hypoxia-reoxygenation for a viability dropout screen. As the readout, cellular viability was measured with CTG assays. (**b**) Effect of hypoxia-reoxygenation on the viability of DMSO-treated control cells (22% reduction in relative viability (*P* < 0.001)). (**c**) Scatter plot of DMSO-normalized relative viability values in normoxia vs. hypoxia-reoxygenation for each drug concentration. Correlation curve depicts the linear model of the correlation of the values between treatments. Horizontal line depicts the average viability of DMSO-treated cells in hypoxia-reoxygenation. Significant viability-improving effects are depicted with red dots for the maximally effective drug concentrations. The dot representing gefitinib is indicated in blue.

To use an independent approach for functional screening of molecular pathways regulating cardiomyocyte survival under hypoxia-reoxygenation, a drug library consisting of 689 FDA-approved compounds was leveraged (Fig. 2a). For the drug library screen, HL-1 cardiomyocytes were cultured in the presence of the compounds at four different concentrations. The cells were either subjected to hypoxia for 48 h followed by 24 h in normoxia, or cultured for the whole 72 h in normoxia. Cellular viability measured using a CTG assay in the end of the experiment was used as the readout. The hypoxia-reoxygenation treatment resulted in 22% reduction in viability in DMSO-treated control cells (Fig. 2b).

Drugs with at least one concentration significantly improving viability in hypoxia-reoxygenation, and not reducing viability in normoxia, were considered as cardioprotective. Relative DMSO-normalized viability values for each drug concentration in normoxia and hypoxia-reoxygenation are represented as a scatterplot in Fig. 2c. Significant viability-improving effects are depicted with red dots for the maximally effective drug concentrations. The analysis identified both previously known as well as several novel, potentially cardioprotective compounds (Supplementary Dataset S3). As examples of clinically used compounds with known cardioporotective activity in ischemia, ramipril (ACE inhibitor) and azilsartan (angiotensin II receptor blocker)^34–36^ were identified as drugs promoting viability of the cardiomyocytes.

### Protein interaction analyses

**Figure 3:**
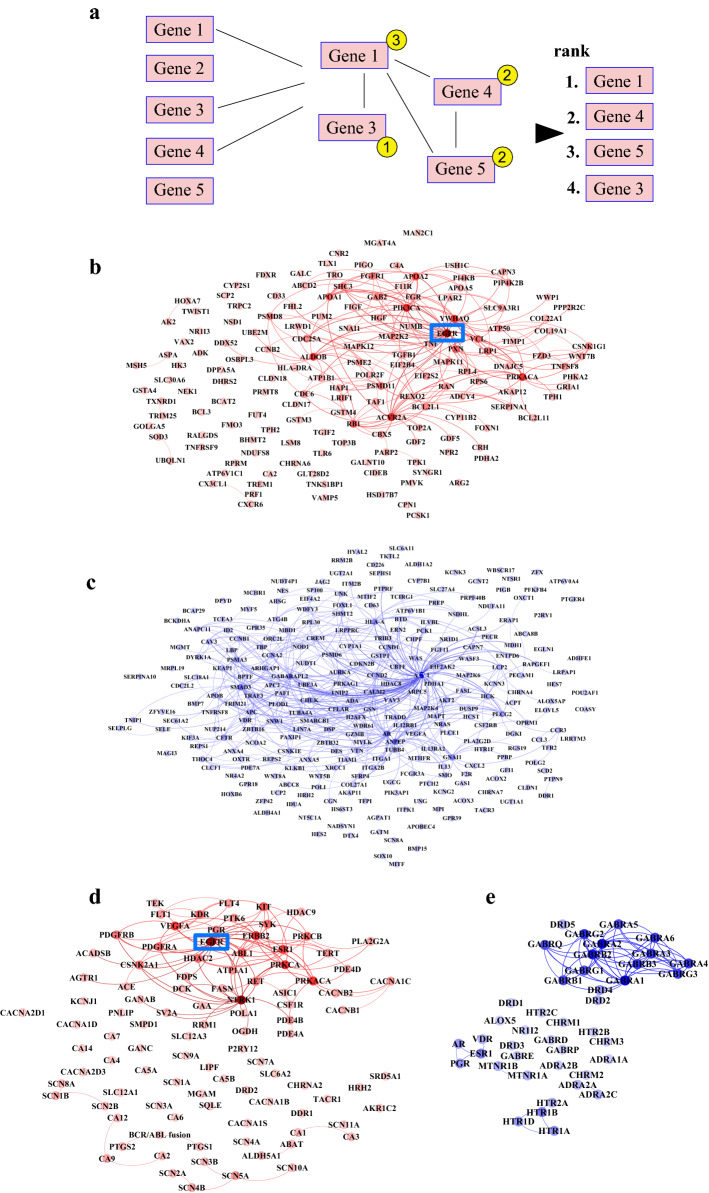
Protein–protein interaction analyses of significant hits from shRNA and drug library screens. (**a**) Schematic outline of the protein–protein interaction analysis. Significant gene or protein target hits from the screens were selected and number of their mutual interactions were calculated (depicted in yellow circles). Targets were subsequently ranked according to the number of their respective interactions. The lists were generated separately for down- and upregulated targets. (**b**–**e**) Significant hits from the screens were analyzed for their mutual interactions based on experimentally validated protein interaction information. Color of each node is scaled according to the quantity of interactions and color of each line corresponds to the node it originates from. (**b**) Genes down-regulated by shRNAs enriched by hypoxia-reoxygenation compared to normoxic controls. (**c**) Genes down-regulated by shRNAs depleted by hypoxia-reoxygenation compared to normoxic controls. (**d**) Protein targets of antagonistic drugs enhancing survival in hypoxia-reoxygenation. (**e**) Protein targets of agonistic drugs enhancing survival in hypoxia-reoxygenation. EGFR is highlighted with a blue rectangle.

To address the putative interactions of the proteins encoded by the genes targeted with the most significantly regulated shRNAs—i.e. proteins that could serve as potential drug targets to promote viability—protein–protein interaction analysis was performed (outlined schematically in Fig. 3a). The analysis was based on experimentally-validated interactions from the PSICQUIC web service. Among genes targeted with upregulated shRNAs, EGFR was the most central node with most mutual primary interactions (Fig. 3b). Interestingly PIK3CA (p110 catalytic subunit of phosphatidylinositol 3-kinase), closely associated with EGFR signaling^37^, was also among the proteins with most interactions. Analysis of proteins encoded by genes targeted with the significantly downregulated shRNAs identified ACY1 (aminoacylase 1), a protein related to urea cycle and amino acid metabolism, as the most interconnected (Fig. 3c). No association of ACY1 with cardiac ischemia–reperfusion has previously been described to our knowledge. AR (androgen receptor) and SMAD3 (mothers against decapentaplegic homolog 3) were also among the highly interconnected proteins. Androgen receptor signaling has been demonstrated to have both detrimental and cardioprotective effects in ischemia–reperfusion injury^38–40^. SMAD3 activity has mainly been implicated in cardiac fibrosis and poor outcome after ischemic injury^41–43^.

Similarly to the shRNA library screen, interactions between the primary protein targets of the drugs that promoted cardiomyocyte survival were analyzed. This was carried out separately for antagonists and agonists (Fig. 3d,e). EGFR—a target for tyrosine kinase inhibitors such as gefitinib and erlotinib—represented the most interconnected node in the analysis for antagonistic drugs (Fig. 3d). Among the targets for agonistic drugs, multiple GABA receptors were included (Fig. 3e) as targets for etomidate.

### Pathways relevant for cardiomyocyte survival under hypoxia-reoxygenation

To comprehensively study the signaling pathways involved in hypoxia-reoxygenation response, data from both the shRNA and drug library screens were applied in a pathway analysis by dimensionality reduction. In the analysis of the shRNA library screen data, GROSS_HIF1A_TARGETS_UP was identified as the most significantly regulated pathway, in accordance with a hypoxic response to the conditions applied for the assay (Table 1). REACTOME_SIGNALING_BY_CONSTITUTIVELY_ACTIVE_EGFR was also included among the significantly regulated pathways, again implying a role for EGFR signaling. Notably, EGFR was also included in the significantly regulated BIOCARTA_AT1R_PATHWAY. In the drug library screen, GO_POSITIVE_REGULATION_OF_MUSCLE_CONTRACTION was the most significantly regulated pathway, while KEGG_ERBB_SIGNALING_PATHWAY was the second most significantly regulated one (Table 2). These findings are in line with the central role of EGFR signaling observed in the protein–protein interaction analysis.

**Table 1:** Significantly regulated pathways in dimensionality reduction-based pathway analysis of the shRNA library screen.

Pathway name	Genes found	Pathway length	P value	P value for category fitness
GROSS_HIF1A_TARGETS_UP	3	8	0.004	0.000
GO_ESTABLISHMENT_OF_ENDOTHELIAL_BARRIER	4	30	0.004	0.043
GNF2_CD14	5	35	0.007	0.035
GO_EPITHELIAL_CELL_CELL_ADHESION	2	14	0.008	0.035
REACTOME_TIGHT_JUNCTION_INTERACTIONS	6	29	0.009	0.009
MCCLUNG_DELTA_FOSB_TARGETS_2WK	5	48	0.009	0.081
GO_POSITIVE_REGULATION_OF_REACTIVE_OXYGEN_SPECIES_BIOSYNTHETIC_PROCESS	5	48	0.012	0.081
GO_NEGATIVE_REGULATION_OF_PEPTIDE_SECRETION	6	49	0.013	0.055
REACTOME_ACETYLCHOLINE_NEUROTRANSMITTER_RELEASE_CYCLE	2	10	0.014	0.010
GO_CLATHRIN_SCULPTED_VESICLE	2	12	0.014	0.021
GO_POSITIVE_REGULATION_OF_PHOSPHOLIPASE_ACTIVITY	5	53	0.015	0.101
COLLER_MYC_TARGETS_UP	3	25	0.018	0.057
PID_IL12_STAT4_PATHWAY	4	33	0.018	0.056
PID_NFKAPPAB_ATYPICAL_PATHWAY	3	17	0.019	0.018
YIH_RESPONSE_TO_ARSENITE_C4	5	18	0.020	0.002
GNF2_MATK	3	25	0.021	0.057
STEIN_ESTROGEN_RESPONSE_NOT_VIA_ESRRA	2	18	0.023	0.070
GO_VENTRICULAR_CARDIAC_MUSCLE_CELL_DIFFERENTIATION	2	19	0.023	0.079
GO_FACE_DEVELOPMENT	6	50	0.024	0.057
GO_BASE_EXCISION_REPAIR	5	39	0.024	0.048
PID_ARF6_PATHWAY	5	35	0.025	0.035
PARK_HSC_AND_MULTIPOTENT_PROGENITORS	6	50	0.025	0.057
GO_REGULATION_OF_CARBOHYDRATE_CATABOLIC_PROCESS	4	42	0.026	0.099
BIOCARTA_AT1R_PATHWAY	4	36	0.027	0.070
GO_VASCULAR_ENDOTHELIAL_GROWTH_FACTOR_RECEPTOR_SIGNALING_PATHWAY	12	74	0.027	0.024
MACLACHLAN_BRCA1_TARGETS_UP	4	21	0.027	0.012
GO_REGULATION_OF_EXTRINSIC_APOPTOTIC_SIGNALING_PATHWAY	15	153	0.027	0.093
BIOCARTA_TID_PATHWAY	3	19	0.030	0.025
PID_IL2_STAT5_PATHWAY	5	30	0.030	0.021
BIOCARTA_THELPER_PATHWAY	3	14	0.031	0.008
GO_VASOCONSTRICTION	3	28	0.031	0.076
GO_STEROID_BINDING	11	91	0.032	0.056
PID_WNT_SIGNALING_PATHWAY	4	28	0.033	0.035
KEGG_DORSO_VENTRAL_AXIS_FORMATION	3	25	0.033	0.057
GO_NEGATIVE_REGULATION_OF_MUSCLE_TISSUE_DEVELOPMENT	5	36	0.033	0.039
GO_STEROL_BINDING	8	43	0.033	0.013
GO_LIPOPOLYSACCHARIDE_MEDIATED_SIGNALING_PATHWAY	4	31	0.034	0.047
GO_REGULATION_OF_PHOSPHATIDYLINOSITOL_3_KINASE_SIGNALING	13	138	0.035	0.101
GO_REGULATION_OF_STEROL_TRANSPORT	6	38	0.038	0.025
REACTOME_GENERATION_OF_SECOND_MESSENGER_MOLECULES	3	27	0.038	0.070
GO_REGULATION_OF_PHOSPHOLIPID_METABOLIC_PROCESS	6	61	0.038	0.092
JEON_SMAD6_TARGETS_DN	4	19	0.039	0.008
GO_REGULATION_OF_CORTICOSTEROID_HORMONE_SECRETION	2	15	0.041	0.043
GO_NEGATIVE_REGULATION_OF_BEHAVIOR	2	17	0.041	0.060
GO_POSITIVE_REGULATION_OF_CIRCADIAN_RHYTHM	2	21	0.041	0.099
GO_POSITIVE_REGULATION_OF_INTERLEUKIN_10_PRODUCTION	3	29	0.042	0.082
REACTOME_RESPONSE_TO_ELEVATED_PLATELET_CYTOSOLIC_CA2_	9	89	0.043	0.087
WANG_RECURRENT_LIVER_CANCER_UP	3	20	0.043	0.031
GO_MOLTING_CYCLE	8	83	0.044	0.096
ST_GAQ_PATHWAY	3	28	0.044	0.076
GO_REGULATION_OF_B_CELL_MEDIATED_IMMUNITY	4	41	0.045	0.094
GO_REGULATION_OF_N_METHYL_D_ASPARTATE_SELECTIVE_GLUTAMATE_RECEPTOR_ACTIVITY	3	15	0.046	0.010
REACTOME_SIGNALING_BY_CONSTITUTIVELY_ACTIVE_EGFR	3	18	0.047	0.021
GO_REGULATION_OF_SHORT_TERM_NEURONAL_SYNAPTIC_PLASTICITY	3	14	0.048	0.008

Pathways with adjusted rounded *P* values ≤ 0.05 and rounded *P* values for category fitness ≤ 0.1 are shown.

**Table 2:** Significantly regulated pathways in dimensionality reduction-based pathway analysis of the drug library screen.

Pathway name	Genes found	Pathway length	P value	P value for category fitness
GO_POSITIVE_REGULATION_OF_MUSCLE_CONTRACTION	7	44	0.042	0.010
KEGG_ERBB_SIGNALING_PATHWAY	5	87	0.043	0.101
GO_REGULATION_OF_PROTEIN_TYROSINE_KINASE_ACTIVITY	6	61	0.043	0.033
GO_REGULATION_OF_CALCIUM_ION_IMPORT	6	103	0.047	0.098
YNTTTNNNANGCARM_UNKNOWN	5	70	0.047	0.066
GO_MUSCLE_CONTRACTION	22	233	0.048	0.036
GO_REGULATION_OF_CATECHOLAMINE_SECRETION	10	43	0.049	0.004
GO_POSTSYNAPSE	26	378	0.049	0.071

Pathways with adjusted rounded *P* values ≤ 0.05 and rounded *P* values for category fitness ≤ 0.1 are shown.

As a potential indication of apoptosis as the activated cell death mechanism, GO_REGULATION_OF_EXTRINSIC_APOPTOTIC_SIGNALING_PATHWAY was detected as a significant pathway in the shRNA library screen (Table 1). Interestingly, lenalidomide and ponalidomide, inhibitors of tumor necrosis factor alpha (TNFα), a central factor in the pathway of extrinsic apoptosis, also demonstrated a moderate improvement in the viability of the cells in the drug library screen (Supplementary Dataset S1).

### Combined analysis of shRNA and drug library screens identifies EGFR as a common hit

The total number of overlapping targets in the shRNA and drug library screenings was 452. Proteins that were identified as potential targets for inhibition in order to stimulate cardiomyocyte survival in both screens included EGFR, Protein Kinase CAMP-Activated Catalytic Subunit Alpha (PRKACA)*,* and carbonic anhydrase 2 (CA2). The drugs included in the drug library screen that were annotated to block the activity of these proteins were: gefitinib, erlotinib, lapatinib, and vandetanib for EGFR; ellagic acid for CA2 and PRKACA; and hydrochlorothiazide for CA2. Proteins that were identified as potential targets for activation in order to stimulate cardiomyocyte survival in both screens were androgen receptor (AR) and vitamin D receptor (VDR). The drugs annotated to increase the activity of these proteins were: levonorgestrel for AR, and alfacalcidol for VDR. The combination analysis thus detected several concordant drug targets from both screens. It is worth noticing, however, that with the exception of gefitinib (annotated only to inhibit EGFR), the drugs may have multiple primary targets.

Taken together, these findings suggest that inhibition of EGFR activity is of potential benefit for promoting cardiomyocyte viability in the condition of hypoxia-reoxygenation in vitro. EGFR has also previously been implicated to regulate cardiac functions in ischemia^44–51^, and several EGFR inhibitor drugs are already in clinical use. Therefore, EGFR was chosen for further experimental validation.

### EGFR inhibitor gefitinib improves cardiomyocyte viability under hypoxia-reoxygenation in vitro

**Figure 4:**
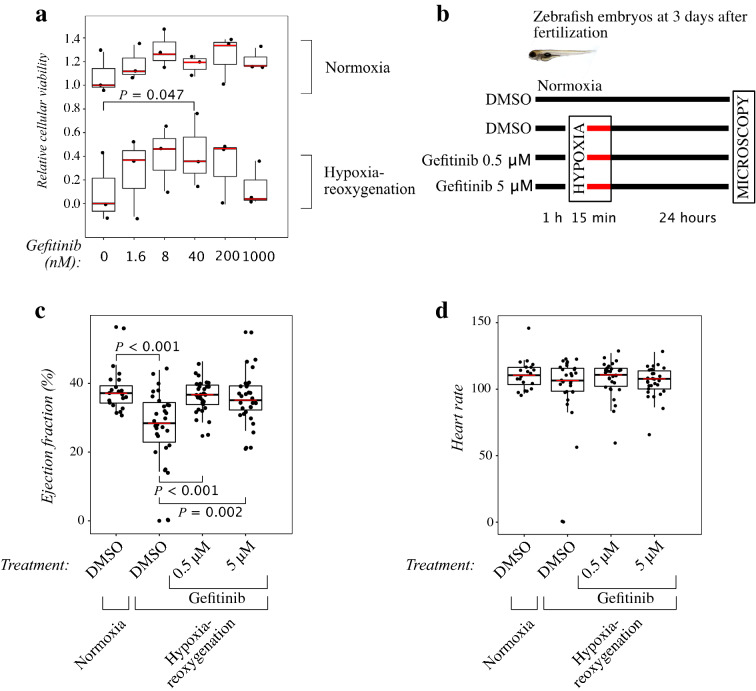
Effects of gefitinib on cardiomyocytes and zebrafish embryos challenged with hypoxia-reoxygenation. (**a**) Cellular viability of HL-1 cells treated with increasing concentrations of gefitinib. Cells were cultured in normoxia or exposed to 24-h hypoxia followed by 24-h reoxygenation. Cellular viability was measured using CTG assays. N = 3. One-way ANOVA followed by paired T-test and FDR-correction of *P* values were used for statistical analyses. Data were normalized by setting the median viability of untreated, normoxic controls to one and median viability of hypoxia-reoxygenation treated control samples to zero. (**b**) Outline of zebrafish embryo experiments. Embryos were treated for 1 h with 0, 0.5 or 5 μM of gefitinib before being exposed to hypoxia for 15 min and reoxygenation for 24 h. (**c**,**d**) Ventricular ejection fraction (**c**) and heart rate (**d**) of zebrafish embryos exposed to 15 min of hypoxia followed by reoxygenation for 24 h. The embryos were treated with 0, 0.5 or 5 μM of gefitinib. N = 20 for embryos in normoxia, n = 30 for all groups treated with hypoxia-reoxygenation. Kruskal–Wallis test followed by Mann–Whitney U test and subsequent FDR-correction of *P* values were used for statistical analyses.

To validate the significance of EGFR inhibition in promoting cardiomyocyte viability, HL-1 cells were cultured in the presence of different concentrations of gefitinib in hypoxia for 24 h, followed by reoxygenation for 24 h. The viability of the cells at the end of the treatment was compared with cells cultured in normoxia. Under normoxic conditions, gefitinib promoted a non-significant trend for increase in cellular viability (Fig. 4a). However, in cells subjected to hypoxia-reoxygenation, 40 nM gefitinib significantly increased the viability of cells (Fig. 4a).

### EGFR inhibitor gefitinib preserves cardiac contractility in a zebrafish model of ischemia–reperfusion injury

To model ischemia–reperfusion injury in vivo, zebrafish embryos from embryonic day three were transferred to deoxygenated water for 15 min and then returned back to regular normoxic conditions for 24-h reoxygenation (Fig. 4b). The protocol reduced the cardiac ejection fraction (EF) by 8.8 percent points, as compared to embryos maintained in normoxia (EF 28.4% *vs.* 37.2%; *P* < 0.001) (Fig. 4c). The protocol was also lethal to two out of the 30 embryos. However, no significant change in the heart rate (HR) was observed (Fig. 4d). Treatment with gefitinib at concentrations of 0.5 or 5 μM preserved the ejection fraction after hypoxia-reoxygenation, as compared to control fish treated with the DMSO solvent alone (Fig. 4c). No significant differences were observed in the heart rate in gefitinib-treated embryos compared to DMSO-treated ones (Fig. 4d).

Longer duration of hypoxic treatment up to 30 min drastically increased lethality of the DMSO-treated embryos up to 67% (16 out of 24 embryos). Lethality in embryos treated with 5 μM gefitinib was 42% (9 out of 21 embryos). The difference in the number of dead and live embryos between DMSO- and gefitinib-treated groups was not, however, statistically significant (*P* = 0.14, Fisher’s exact test). Ejection fraction was also significantly reduced by the treatment in 30 min of hypoxia followed by 24 h of reoxygenation, as compared to embryos maintained in normoxia (Supplementary Fig. S2A). Again, treatment with gefitinib at 5 μM significantly preserved the ejection fraction in hypoxia-reoxygenation (Supplementary Fig. S2A). Significant differences were also observed in heart rate between the embryos subjected to hypoxia-reoxygenation vs. normoxia, while there was no statistically significant difference between the DMSO-treated and gefitinib-treated embryos undergoing the hypoxia-reoxygenation (Supplementary Fig. S2B). However, any conclusions drawn from these experiments need to take into consideration that for several of the embryos the ejection fraction or the heart rate was 0 and thus reflected also the mortality associated with the protocol.

To further analyze the effects of hypoxia-reoxygenation procedure on embryonic hearts, whole-mount immunofluorescence analyses of the myocardium were carried-out (Supplementary Fig. S2C,D). As expected, thinner myocardial layers were observed 24 h after hypoxia-reoxygenation in the DMSO-treated embryos as compared to embryos maintained in normoxia, consistent with cell loss within the myocardium. The myocardium of gefitinib-treated embryos suffered less from the hypoxia-reoxygenation procedure and the myocardial layer was significantly thicker as compared to DMSO controls (Supplementary Fig. S2C,D).

### Pan-ErbB inhibitor afatinib does not preserve cardiac contractility in a zebrafish model of ischemia–reperfusion injury

Two other receptors belonging to the same ErbB gene family as EGFR–ErbB2 and ErbB4—have previously been documented to be necessary for normal development and maintenance of the myocardium^52,53^. These previous findings imply that targeting of ErbB2 or ErbB4 would not represent a rational strategy for protecting cardiac functions during ischemia–reperfusion, similar to proposed here for targeting of EGFR with gefitinib. To experimentally address whether inhibition of EGFR produces different results from inhibition of other ErbB receptors in the zebrafish model, the effects of gefitinib were compared to the effects of afatinib, a clinically approved pan-ErbB inhibitor blocking the activity of all the three kinase-competent ErbB family members: EGFR, ErbB2 and ErbB4. As expected, and differently from gefitinib (Supplementary Fig. S2A,B), treatment with afatinib did not rescue either the ejection fraction or the heart rate from the modest drop induced by a short hypoxia-reoxygenation treatment (Supplementary Fig. S2E,F). In contrast, afatinib actually promoted a significant further reduction in both ejection fraction and heart rate when compared to control embryos maintained in normoxia (Supplementary Fig. S2E,F). In concordance with the in vivo results, afatinib caused a prominent reduction in cellular viability in the in vitro drug library screen, both in normoxia and in hypoxia-reoxygenation (Supplementary Dataset S1). Taken together, these findings indicate that EGFR, but not ErbB2 or ErbB4, serves as a target for improving cardiomyocyte viability and function after hypoxia and subsequent reoxygenation.

### Signaling pathways associated with EGFR signaling and hypoxia-reoxygenation

**Figure 5:**
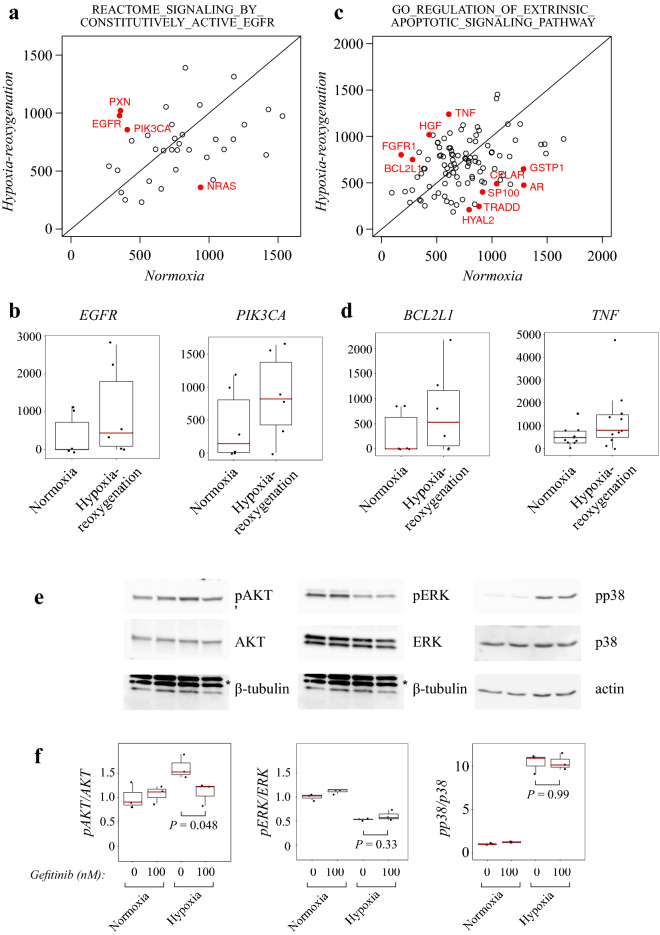
Pathways involved in hypoxia-reoxygenation response and EGFR-mediated signal transduction. (**a**) Scatterplot of mean reads of shRNAs (normoxia *vs.* hypoxia-reoxygenation) against genes in the pathway REACTOME_SIGNALING_BY_CONSTITUTIVELY_ACTIVE_EGFR. Significantly differentially expressed genes are depicted with red dots and labels. (**b**) Boxplots of reads for shRNAs against *EGFR* and *PIK3CA*. (**c**) Scatterplot of mean reads of shRNAs against genes in the pathway GO_REGULATION_OF_EXTRINSIC_APOPTOTIC_SIGNALING_PATHWAY. Significantly differentially expressed genes are depicted with red dots and labels. (**d**) Boxplots of reads for shRNAs against *BCL2L1* and *TNF*. (**e**) Western analysis of phosphorylated and total AKT, ERK and p38 in HL-1 cells treated overnight with 0 or 100 nM of gefitinib and exposed to hypoxia for 3 h. β-Tubulin (the lowest band) or actin were used as loading controls, * = non-specific bands. (**f**) Densitometric analysis of phosphorylated AKT, ERK and p38 against total protein levels (arbitrary units). N = 3 biological replicates. One-way ANOVA followed by paired T-test was used for statistical comparisons.

To further examine the pathways and signaling mechanism involved in hypoxia-reoxygenation injury and EGFR-mediated signal transduction, two significant pathways from the pathway analysis in shRNA screen (Table 1) were analyzed in detail. Reads of shRNAs against the members of the REACTOME_SIGNALING_BY_CONSTITUTIVELY_ACTIVE_EGFR pathway are visualized as a scatterplot (mean reads in normoxia *vs*. hypoxia-reoxygenation) in Fig. 5a. Genes of significantly differentially expressed shRNAs are visualized in red dots. *PIK3CA*, encoding the catalytic subunit of phosphatidylinositol 3-kinase (PI3K), a central downstream signal mediator of EGFR, was among the significant hits, in addition to *EGFR*. Concordantly, shRNAs against *PIK3CA* were enriched in hypoxia-reoxygenation, suggesting the benefit of suppression of PI3K signaling. Normalized sequencing reads of individual shRNAs targeting *EGFR* or *PIK3CA* genes are visualized as boxplots in Fig. 5b.

As mentioned above, the pathway GO_REGULATION_OF_EXTRINSIC_APOPTOTIC_SIGNALING_PATHWAY was another significant finding in the pathway analysis, indicating apoptosis as the activated mechanism of cell death in the screening setup (Fig. 5c). Reads of shRNAs targeting *BCL2L1* (encoding anti- and pro-apoptotic proteins BCL-X_L_ and BCL-X_S_, respectively)^54^ or *TNF* are visualized in Fig. 5d. Suppression of these genes, and presumably apoptosis in general, is potentially and logically beneficial in hypoxia-reoxygenation conditions. Interestingly, it has been demonstrated that EGFR and TNFα can cross-activate each other^55^.

Activities of downstream signaling proteins of EGFR were analyzed by Western blotting after cells were maintained for 3 h in hypoxia in the presence or absence of gefitinib. A clear increase in the level of phosphorylated AKT and p38 and a decrease in the phosphorylation of ERK were detected in response to hypoxia (Fig. 5e,f). Reoxygenation after hypoxia led to rapid normalization of differences observed at the phosphorylation level, and the condition was not studied further. Noteworthly, *MAPK11* and *MAPK12*, genes encoding p38 isoforms, were among the hits in shRNA screen where shRNAs against them were enriched (Fig. 3a). In accordance with the shRNA screen data, in which shRNAs against the catalytic PI3K subunit were enriched in hypoxia-reoxygenation, AKT phosphorylation (a direct target of PI3K) was decreased by EGFR inhibition with gefitinib (100 nM overnight) (*P* = 0.048) (Fig. 5e,f, left panels), returning the phosphorylation level close to the level observed in normoxia. No differences in ERK and p38 phosphorylation after gefitinib treatment were detected. These findings are consistent with a role for PI3K-AKT signaling in mediating the effect of gefitinib during hypoxia-reoxygenation.

## Discussion

Here, we screened for novel therapeutic targets for myocardial ischemia–reperfusion injury using a combination of systematic functional screens based on shRNA and drug libraries in cardiomyocytes subjected to experimental hypoxia-reoxygenation. Concordant putative targets that were identified with both approaches included *AR*, *CA2*, *EGFR*, *PRKACA* and *VDR*. EGFR emerged as a putative novel therapeutic target as it was (1) found to be enriched in pathway analyses of both screens, (2) its inhibition with the EGFR-selective TKI gefitinib enhanced the viability of cultured HL-1 cardiomyocytes in hypoxia-reoxygenation in vitro, and (3) its inhibition with gefitinib, but not with the more wide-spectrum ErbB inhibitor afatinib, preserved the cardiac contractility of zebrafish embryos exposed to ischemia–reperfusion injury in vivo.

Our approach was validated by the identification of several previously known cardioprotective targets or compounds. For example, *AKT2*, a gene encoding a known pro-survival signaling protein^56^, was detected in the shRNA screen. Moreover, ACE and angiotensin receptor blocking agents (ramipril and azilsartan, respectively), were detected as cardioprotective compounds in the drug library screen. The potential novel targets that were concordantly identified in both screens included *AR*, *CA2*, *EGFR*, *PRKACA*, and *VDR*. Ellagic acid, an inhibitor of *PRKACA* and *CA2* that has multiple additional targets according to the Drug Gene Interaction Database (DGIdb)^19^, has also previously been shown to have cardioprotective potential^57^, while the direct involvement of *PRKACA* or *CA2* in cardiomyocyte survival has not been previously addressed. The cardioprotective drug regulating AR that was included in the drug library used was the progesterone receptor (PGR) agonist levonorgestrel, with agonistic activity also for AR, and antagonistic activity for estrogen receptor (ESR) as annotated in DGIdb^19^. Consistently, ethynodiol acetate, another PGR agonist, as well as fulvestrant, an ESR antagonist were also detected as a cardioprotective hits in the drugs screen. Finally, flutamide, an AR antagonist, was found out to drastically reduce the viability of cardiomyocytes at 5 μM concentration, while other AR antagonists, drospirenone and bicalutamide did not significantly affect the viability (Supplementary Dataset S1). Taken together, these observations indicate that AR and PGR are potential druggable targets for myocardial ischemia, while their pharmacological targeting can be complicated due to drug compounds’ activities towards other sex hormone receptors. These considerations may explain some of the controversial results in earlier preclinical studies on AR targeting in cardiac ischemia–reperfusion injury^38–40^.

Both the RNA interference-based and the pharmacological screens indicated cardioprotective potential for EGFR inhibition. Moreover, cardioprotective effect of EGFR inhibition with gefitinib was suggested by experimentation both using HL-1 cells in vitro as well as in zebrafish embryos in vivo. EGFR (ErbB1) is a receptor tyrosine kinase that belongs to the four-member gene family of EGFR/ErbB receptors. ErbB receptors have been demonstrated to regulate embryonic development of the heart^52,53,58,59^, as well as the development of cardiac diseases, such as ischemia^60–63^. For example, *Erbb2* and *Erbb4* knockout mouse embryos die with defects in the development of cardiac trabeculae^52,53^, and *Egfr* null mice demonstrate defects in cardiac valve development and develop cardiac hypertrophy^59^. *EGFR* mRNA expression has also been reported to be increased in H9c2 rat cardiomyoblasts during hypoxia-reoxygenation^64^, as well as in chronically ischemic human heart in patients undergoing coronary artery bypass grafting^63^. However, in our own earlier study of mRNA expression data from a publicly available database, decreased *EGFR* expression was observed in acute myocardial infarction^65^, possibly reflecting differential regulation of EGFR expression during different stages of cardiac ischemia.

Consistent with results presented here, several lines of previous evidence indicate that EGFR activity is detrimental for the survival or function of cardiomyocytes. Gefitinib has been shown to reduce isoproterenol-induced cardiac hypertrophy and apoptosis as well as decrease of heart contractility in the mouse^66^. Chemical EGFR inhibition has also been demonstrated to protect from ischemia–reperfusion injury in type 2-diabetic rats^48^. Moreover, EGFR activation by the ligand amphiregulin has been shown to contribute to cardiac fibrosis and loss of contractility after infarction in mice^49^. Additionally, chemical EGFR inhibition as well as RNAi-mediated EGFR knockdown have been shown to prevent post-ischemic arrhythmias in rats^50^. Finally, cetuximab, an EGFR-targeting antibody, prevents cardiac rupture and post-infarction mortality in TIMP3-knockout mice in which EGFR signaling is enhanced^51^. Importantly, EGFR targeting therapies have not been tested in clinical trials in humans diagnosed with myocardial infarction.

In contrast to our observations, there is also abundant contradictory preclinical evidence indicating a protective role for EGFR signaling in the survival of cardiomyocytes during myocardial ischemia and in other cardiac stresses^67^. For example, in response to chronic catecholamine stimulation in mice, EGFR transactivation by β1-adrenergic receptor has been shown to prevent cardiomyocyte apoptosis and to protect from reduction of cardiac contractility^68^. This was demonstrated by a 2-week treatment with erlotinib in combination with isoproterenol, which together increased cardiomyocyte apoptosis and reduced the fractional shortening, while there was no effect when erlotinib or isoproterenol were administered alone^68^. EGFR activity has also been demonstrated to be necessary for the prevention of ischemic injury by ischemic preconditioning and adenosine in mice^45^. Similarly, prevention of ischemic injury with bradykinin in isolated rat hearts depends on signaling through EGFR^46^. Additionally, administration of the EGF ligand has been reported to improve ischemia–reperfusion tolerance in isolated hearts of type 1-diabetic rats^44^ and in isolated mouse hearts^47^. Although generally considered non-cardiotoxic in human, treatment with gefitinib has been associated with a case of vulnerable plaque rupture in the context of recurrent myocardial infarction^69^ and a case of reversible cardiomyopathy^70^. Preclinically, cardiotoxicity of gefitinib has been studied in H9c2 cardiomyoblasts and rat models, in which significant toxicities were noticed only at relatively high concentrations (5 μM and greater)^71^. It has also been shown in a rat model, that gefitinib-induced cardiotoxicity can be attenuated with liraglutide^72^.

While the previous preclinical studies have frequently tested the effects of EGFR inhibition by the TKIs such as erlotinib or AG1478, to our knowledge gefitinib has not been been tested for the treatment of myocardial ischemia. Morever, in accordance with our observations with the pan-ErbB inhibitor afatinib, the more wide-spectrum ErbB TKIs, such as AG1478, may not possess similar cardioprotective potential as selective EGFR inhibition. Additionally, concomitant disease states such as diabetes might affect the advantage of EGFR inhibition on cardioprotection^67^.

Pathway analyses identified EGFR signaling and extrinsic apoptotic signaling as significant in the hypoxia-reoxygenation setting. Interestingly, the two pathways have been reported to interact at the level of molecular cross-talk between TNFα and EGFR^55^. The PI3K-AKT axis was also identified as a potential mediator of the effect of gefitinib. While AKT is a well-known survival factor in various biological models, its activity has also been shown to predispose cells to oxidative apoptosis^73^. Interestingly, in our Western analyses, gefitinib restored the induced level of AKT phosphorylation in hypoxia close to the level observed in normoxia, possibly reflecting a block in a hypoxia-induced stress response. The exact molecular mechanisms of the effects of EGFR inhibition by gefitinib that promote cardiomyocyte surval in hypoxia, however, remain to be elucidated in future studies.

Both the screening and validations were performed using HL-1 cells, an immortalized atrial cardiomyocyte line, which has its limitations as a cardiomyocyte model^74^. These findings should be further validated using primary cardiomyocytes or human iPSC-derived cardiomyocytes. Our in vivo model on zebrafish embryos recapitulated the in vitro observations suggesting cardioprotective effects for gefitinib. The effect of gefitinib was particularly robust in the longer, 30-min ischemia-exposure where gefitinib conferred an overall survival benefit, in addition to preserving the cardiac contractility. It is worth noticing that the zebrafish heart is capable of regenerating^75,76^ representing a profound difference between the model and the human cardiac system. Thus, interpreting the significance of data obtained using zebrafish for human biology is challenging. However, the zebfrafish model has previously been successfully exploited to predict cardiotoxicity of the TKIs sunitinib and sorafenib^77^. Interestingly, gefitinib was found not to be cardiotoxic in the same analyses^77^.

Taken together, these results warrant further testing of EGFR-selective TKIs, such as gefitinib, as therapeutics in mammalian models of myocardial ischemia.

## Supplementary Information

Supplementary Information 1.

Supplementary Information 2.

Supplementary Information 3.

Supplementary Information 4.

## Data Availability

All data generated or analysed during this study are included in this published article (and its Supplementary Information files).
