# Supplementary material for: Combined genetic and chemical screens indicate protective potential for EGFR inhibition to cardiomyocytes under hypoxia
Source: Sci Rep. 2021 Aug 17;11:16661. doi: 10.1038/s41598-021-96033-z (PMC8371130; doi:10.1038/s41598-021-96033-z)
Supplement: Supplementary file 1 — Supplementary Information 1. [file 41598_2021_96033_MOESM1_ESM.pdf]

Combined genetic and chemical screens indicate protective potential for EGFR inhibition to cardiomyocytes under hypoxia – Supplementary information

Juho Heliste[1,2], Anne Jokilammi[1,3], Katri Vaparanta[1,2,3,4], Ilkka Paatero[3], and Klaus Elenius[1,3,4,5]

[1]Institute of Biomedicine, University of Turku, Kiinamyllynkatu 10, FI-20014 Turku, Finland

[2]Turku Doctoral Programme of Molecular Medicine, University of Turku, Turku, Finland

[3]Turku Bioscience Centre, University of Turku and Åbo Akademi University, Tykistökatu 6, FI-20520 Turku, Finland

[4]Medicity Research Laboratories, University of Turku, Tykistökatu 6, FI-20520 Turku, Finland

[5]Department of Oncology, Turku University Hospital, PO Box 52, FI-20521 Turku, Finland

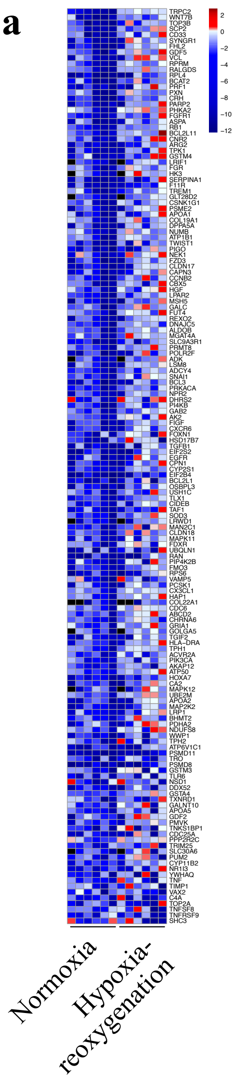

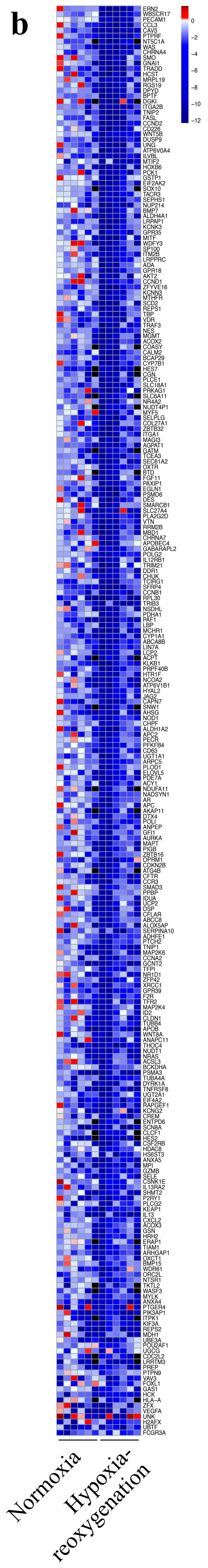

Supplementary Figure S1. Heatmaps of significantly enriched or depleted shRNAs during hypoxia-reoxygenation of HL-1 cells.

Relative abundances of significantly enriched (A) or depleted (B) shRNAs during hypoxia-reoxygenation treatment in normoxic and hypoxia-reoxygenation samples. ShRNA reads for individual shRNAs (in columns) per each gene (in rows), were normalized to their abundance in the original shRNA plasmid pool. The normalized values were log2-transformed to make fold changes symmetrical around 0.

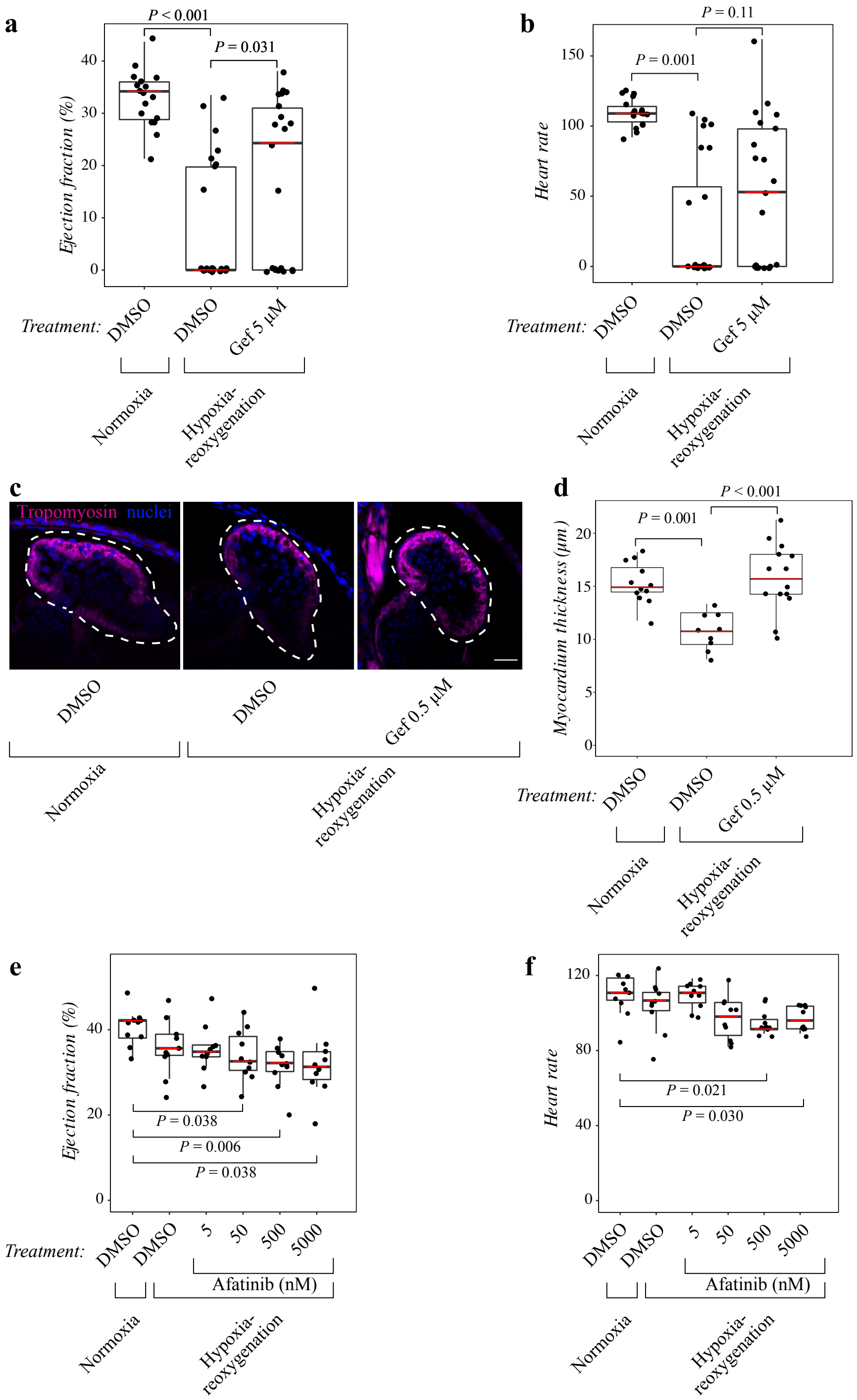

Supplementary Figure S2. Effect of gefitinib and afatinib on cardiac functions and morphology of zebrafish embryos during hypoxia-reoxygenation.

A-B: Ventricular ejection fraction (A) and heart rate (B) of zebrafish embryos exposed to 30-minute hypoxia in deoxygenated water, followed by reoxygenation in normoxia for 24 hours. The embryos were treated with DMSO or gefitinib at a concentration of 5 µM added to the water one hour prior to the start of the hypoxic exposure. n = 17 for embryos in normoxia, n = 24 for DMSO-treated embryos in hypoxia-reoxygenation and n = 21 for gefitinib-treated embryos in hypoxia reoxygenation.

C-D: Immunofluorescence analysis of whole-mount zebrafish embryos. C) Confocal images of embryonic zebrafish heart after hypoxia-reoxygenation experiment. The ventricle of the heart is outlined with white dashed line. The contrast and brightness of images were individually and linearly adjusted to ensure comparable visualisation of nuclei (stained with DAPI) and myocardium (stained with anti-tropomyosin) between different samples. Scale bar, 25 μm. D) Quantification of the thickness of myocardium. n = 12 for embryos in normoxia, n = 9 for DMSO-treated embryos in hypoxia-reoxygenation and n = 14 for gefitinib treated embryos in hypoxia-reoxygenation.

E-F: Ventricular ejection fraction (E) and heart rate (F) of zebrafish embryos exposed to 15-minute hypoxia in deoxygenated water, followed by reoxygenation in normoxia for 24 hours. The embryos were treated with DMSO or afatinib at concentrations of 5, 50, 500 or 5000 nM added to the water one hour prior to the start of the hypoxic exposure. n = 10 for each group. Kruskal-Wallis test followed by Mann-Whitney U test, or one-way ANOVA followed by Student's t-test, and subsequent FDR-correction of *P*-values were used for statistical analyses.

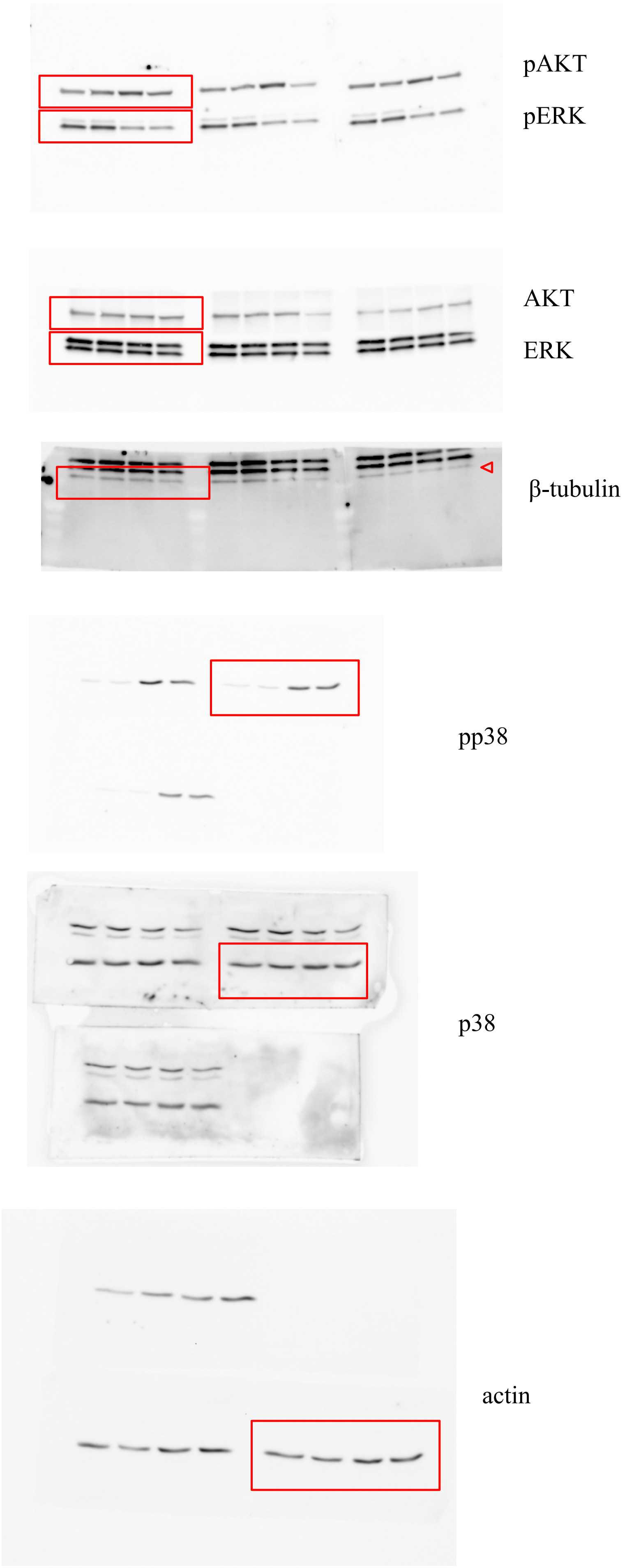

Supplementary Figure S3. Whole Western blots used to prepare Figure 5.

Supplementary Datasets

Supplementary Dataset S1. Compounds included in the drug library screening and raw viability data.

Supplementary Dataset S2. Copy numbers of barcoded shRNAs in the used plasmid library and in HL-1 cells with or without hypoxia-reoxygenation treatment.

Data are presented both as normalized to 20 million reads in each condition and as raw NGS data.

Supplementary Dataset S3. Drugs included in the library that were found to promote survival during hypoxia-reoxygenation.
